# First observation of a leucistic bearded capuchin monkey (*Sapajus libidinosus*)

**DOI:** 10.1007/s10329-026-01243-6

**Published:** 2026-02-08

**Authors:** Tiago Falótico, Tatiane Valença

**Affiliations:** 1 Neotropical Primates Research Group, São Paulo, Brazil; 2 Max Planck Institute for Evolutionary Anthropology, Leipzig, Germany; 3 Institute of Psychology, University of São Paulo, São Paulo, Brazil; 4 Max Planck Institute for Animal Behavior, Konstanz, Germany

**Keywords:** Leucism, Neotropical primates, Pigmentation, Fur color, Genetic mutations, Polymorphism

## Abstract

Leucism, a pigmentation disorder resulting in partial loss of coloration, is rare in neotropical primates. This manuscript presents the first report of a leucistic robust capuchin monkey (*Sapajus libidinosus*), observed at Ubajara National Park, Brazil. Our findings contribute to the sparse literature on leucism in New World monkeys, emphasizing the importance of long-term studies for monitoring such rare anomalies.

**Supplementary Information:**

The online version contains supplementary material available at 10.1007/s10329-026-01243-6.

## Introduction

Leucism is a congenital disorder causing partial or total loss of melanin in animal fur or feathers, though eye color remains normal (Acevedo and Aguayo 2008). It can be caused by mutations in several genes (Jacinto et al. 2025) and can be influenced by factors such as poor habitat, inadequate diet (Owen and Skimmings 1992), and pollution (Møller and Mousseau 2001). Leucistic animals may have lower survival rates because predators and hunters can spot them more easily (Owen and Skimmings 1992; Peles et al. 1995).

While leucism is well-documented in birds and some mammals, reports in primates are exceedingly rare (Ramos-Luna et al. 2022). In platyrrhines, most of those reports are for the genera *Callithrix* and *Alouatta* (Aximoff et al. 2020; Ramos-Luna et al. 2022). There is also one report for *Ateles* (Ramos-Luna et al. 2022), and one for *Cebus capucinus imitator* from an island population (Duquette et al. 2015).

Robust capuchin monkeys (*Sapajus* spp.) have a wide variation of color, from dark brown to light golden yellow coats, but their arms, legs, and tail are darker compared to the rest of the body, usually dark brown or black, except for the blonde capuchins (*S. flavius*), which have golden yellow arms, legs and tail (Roosmalen and Roosmalen 2016). None of the *Sapajus* species has a whitish fur color.

Reports of anomalous pigmentation in robust capuchin monkeys (*Sapajus*) are rare or underreported. There is a report of albinism in *Sapajus nigritus* in captivity (Bicca-Marques 1988), and another in *Sapajus apella* in captivity (Henriques et al. 2019); however, there is no report of leucism.

Here, we present the first report of a leucistic robust capuchin monkey (*Sapajus libidinosus*) observed in situ, discuss its distinguishing features, and consider the implications for the individual and population.

## Materials and methods

Field observations were conducted in Ubajara National Park, Ceará state, Brazil. The park spans approximately 6300 hectares and encompasses montane forests, caves, and waterfalls. It has a subtropical climate and supports a population of bearded capuchin monkeys (*S. libidinosus*) that has been studied since 2020 (Falótico et al. 2022, 2024a, b; Valença and Falótico 2023; Valença et al. 2024, 2025).

Video recordings and GPS points were taken using a mobile phone (Apple iPhone 16 Pro) in the first observation and a camcorder (Canon Vixia HF R52) in the subsequent observations. 

## Results

**Fig. 1:**
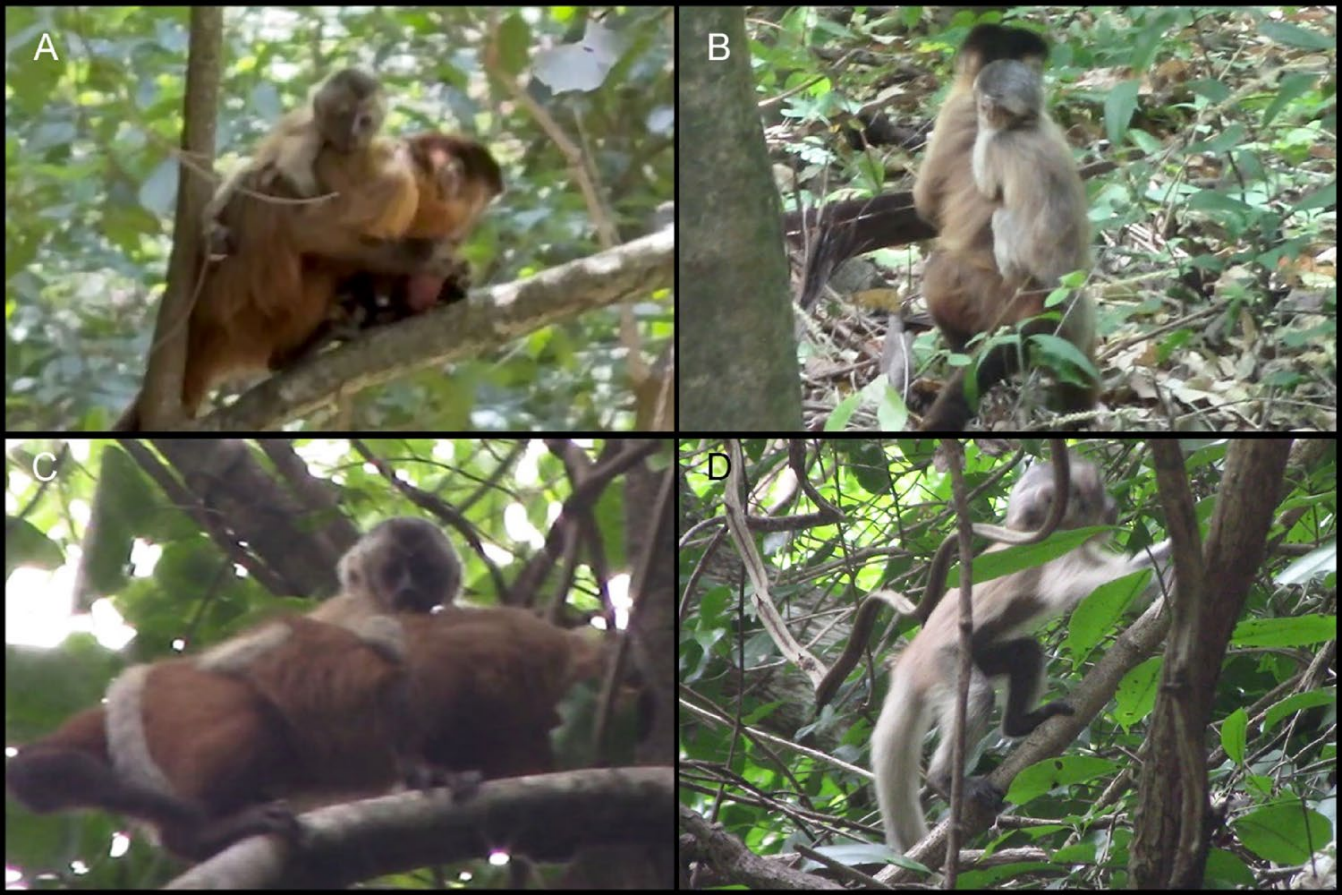
Leucistic infant individual of bearded capuchin monkey (*Sapajus libidinosus*) from Ubajara National Park, and its mother. Note limbs and tail with whitish coloration; for reference, see the mother’s normal fur color pattern. Photograms from videos from the Neoprego database

While performing an automated sound recording setup in the target study group’s area on the 23rd of August 2025, by 11:50 a.m., TF encountered a neighboring group that lives in the same area (3° 49′ 38.28” S; 40° 54′ 0.36′′ W) and sometimes meets with the study group. He observed an adult female carrying a male infant with an atypical fur color pattern (Fig. 1A).

**Fig. 2:**
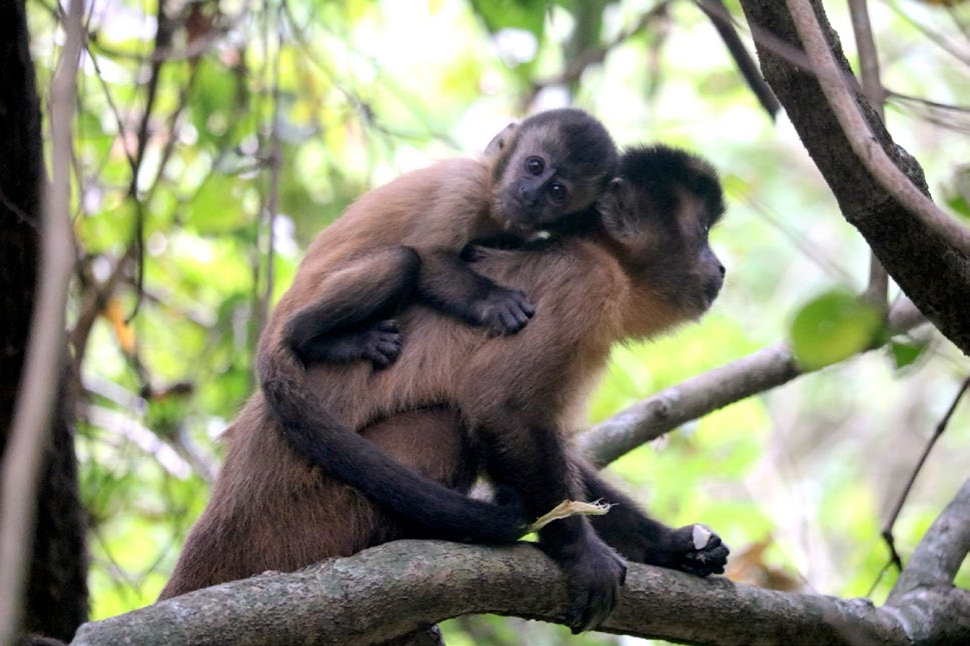
A typical infant of the same population and similar age, showing the normal color pattern. Note the dark brown tail and forearm compared to the leucistic individual in Fig. 1. Photo by TV

The infant, approximately three months old, was observed for about 10 min, and it was noted to have whitish arms, legs, and tail. Leucism was suspected based on the pale fur, in contrast to the typical dark brown and black fur on the distal extremities of the limbs and tail of *S. libidinosus* species (Fig. 2). The top of the head presented a more typical brown color, but the back of the head had whitish streaks (Fig. 1B), possibly indicating a partial leucism. The hands and feet exhibited normal black pigmentation, and ocular pigmentation appeared to be normal, which was used to distinguish leucism from albinism (Abreu et al. 2013). No injuries or signs of ill health were observed. Moreover, the leucistic monkey appeared to be neither ostracized nor treated aggressively by group members during the time we observed it, and it was being carried by an adult female, probably its mother.

The same individual was observed again 30 days later (Fig. 1B–D), and we confirmed the same pattern observed previously. He was mostly being carried by an adult female, but was also observed exploring the environment alone (Supplementary Video 1).

**Fig. 3:**
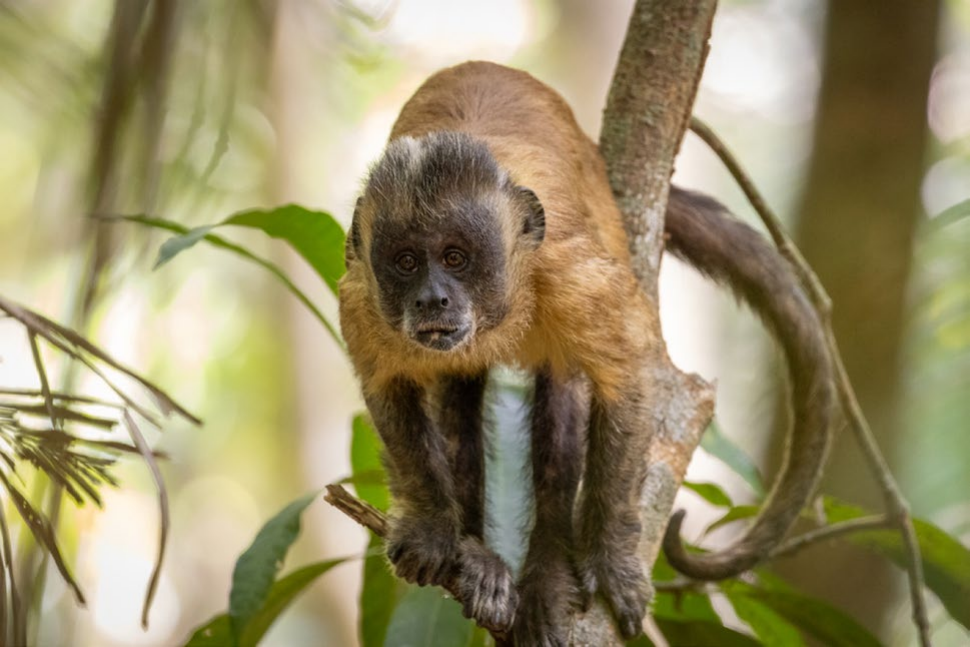
Adult male with a lighter patch of fur on the top of his head. Photo by TF

When looking for past information about the group in older records and videos in our database from past projects (Valença and Falótico 2023; Falótico et al. 2024b; Valença et al. 2024, 2025), we noted that an adult male, potentially the dominant male of another group, also presented an abnormal coloration, albeit subtler. This individual has a whitish patch on the fur on the top of his head on the right side (Fig. 3).

No other individual with those patterns was observed by our team in this population since the beginning of our research in 2020.

The infant was video recorded (Supplementary Video 1), which provided visual confirmation of the leucistic condition and will aid future comparative analysis.

## Discussion

Leucism may result from mutations that affect melanin synthesis or distribution (Jacinto et al. 2025), although diet (Owen and Skimmings 1992), and pollution (Møller and Mousseau 2001) can also influence fur color. Pigmentation anomalies can influence individual fitness, primarily through altered camouflage, social perception, and susceptibility to predation and hunting (Owen and Skimmings 1992; Peles et al. 1995). However, in Ubajara National Park, where the population has a certain degree of protection and human activity is restricted, the survival disadvantage due to hunting may be minimal.

Primates with color patterns that differ from the typical ones for the group can sometimes attract aggression from conspecifics (Leroux et al. 2021). However, we noted no aggression directed at the infant, suggesting that coloration does not strongly mediate group cohesion in *Sapajus* monkeys, at least in this context. Other species of capuchin, the blond capuchin (*S. flavius*), is much lighter in color and potentially hybridizes naturally with *S. libidinosus* (Martins et al. 2023), also suggesting that color patterns do not significantly affect the social interactions in *Sapajus*.

While the presence of one leucistic individual does not necessarily suggest an imminent risk to population health and genetic variability, the presence of two individuals in the same population with abnormal color patterns warrants monitoring to detect potential increases in anomalous phenotypes, which could signal genetic bottlenecks or habitat fragmentation. Ubajara National Park’s well-preserved habitats are likely to support viable primate populations, despite potential negative recessive traits, but vigilance is required as anthropogenic pressures intensify.

Regarding a genetic cause, other than a new mutation or recessive gene expression, a hybridization with *S. flavius* could be the cause of the color variance through introgression of new genes in the population; however, the infant is much lighter and whiter than the pale gold observed in *S. flavius*. Moreover, there were no sightings of adult individuals with the *S. flavius* phenotype in the area, and so far, only this individual with an abnormal color has been observed, suggesting a genetic mutation or rare recessive gene expression as a more likely explanation.

The hypothesis that poor diet or pollution causes the anomalous color lacks strong support at this time, as only two individuals have been observed with an anomalous fur pattern, and a more widespread effect would be expected. However, the population should be carefully monitored, especially if other individuals are spotted with anomalous fur color in different groups in the region.

This study documents the first observation of leucistic robust capuchin monkeys, advancing our understanding of rare pigmentation disorders in wild primates. The individuals described here were apparently healthy, socially integrated, and exhibited behaviors typical of the species. Further research, including genetic analyses and long-term population monitoring, is essential to determine the prevalence and implications of leucism in capuchin monkeys and other Neotropical primates.

## Supplementary Information

Below is the link to the electronic supplementary material.

Supplementary Material 1

## Data Availability

All data supporting the findings of this study are included within this paper and its Supplementary Information. The complete video recordings are available from the corresponding author upon request.
